# A Case of Device Closure of an Eccentric Atrial Septal Defect Using a Large Device

**DOI:** 10.7759/cureus.27447

**Published:** 2022-07-29

**Authors:** Ramachandra Barik, Rudrapratap Mahapatra

**Affiliations:** 1 Cardiology, All India Institute of Medical Sciences, Bhubaneswar, Bhubaneswar, IND; 2 Cardiothoracic Surgery, All India Institute of Medical Sciences, Bhubaneswar, Bhubaneswar, IND

**Keywords:** right atrial and superior vena junction injury, device closure, large device, eccentric location, atrial septal defect

## Abstract

Device closure of an eccentric atrial septal defect can be challenging and needs technical modifications to avoid unnecessary complications. Here, we present a case of a 45-year-old woman who underwent device closure of an eccentric defect with a large device. The patient developed pericardial effusion and left-sided pleural effusion due to injury to the junction of right atrium and superior vena cava because of the malalignment of the delivery sheath and left atrial disc before the device was pulled across the eccentric defect despite releasing the left atrial disc in the left atrium in place of the left pulmonary vein. These two serious complications were managed conservatively with close monitoring of the case during and after the procedure.

## Introduction

Device closure is recommended as class I and level B indication for an ostium secundum atrial septal defect (OS ASD) with suitable anatomy without significant pulmonary arterial hypertension [1]. The anatomy of the atrial defect is the most important factor when deciding between surgery and transcatheter closure [2]. Device closure of an eccentric OS ASD eccentric defect with a large device is challenging and needs technical modifications to avoid unnecessary complications [3-5]. When the ASD has malaligned, eccentric or deficient rims, then the wiring of the left upper pulmonary vein, negotiation of the sheath and dilator and the release of the left atrial disk are challenging and technical modifications are needed for the successful deployment of the device [5].

This case was previously presented as an e-poster titled "Device closure of an eccentric atrial septal defect using a large device" at CSI Frankfurt 2022 (June 22-25, 2022).

## Case presentation

**Figure 1:**
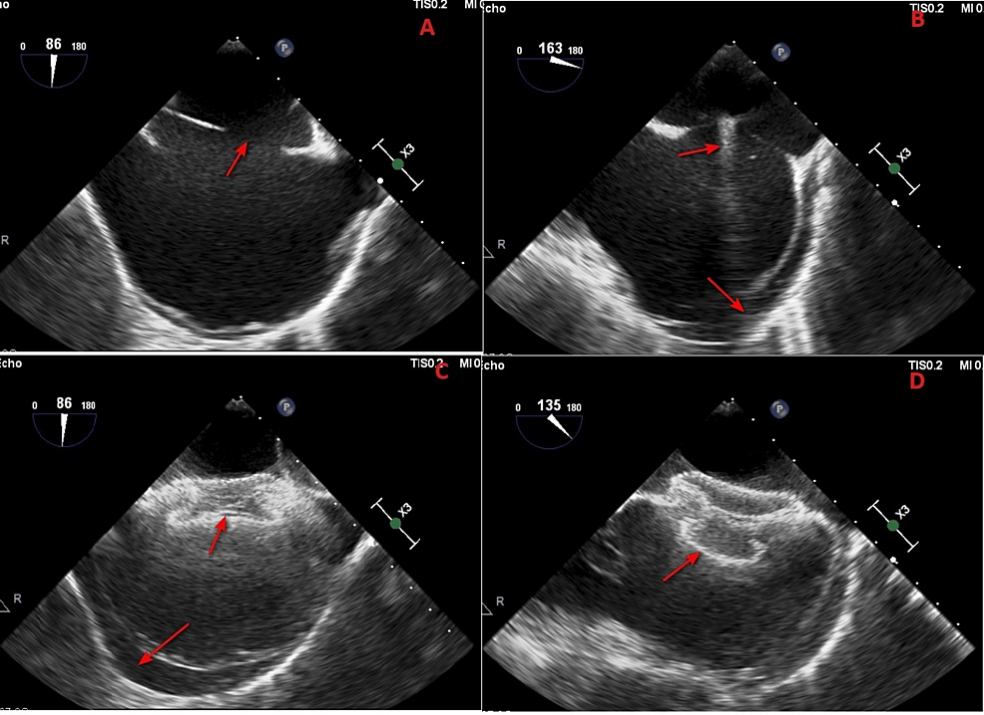
Transesophageal echocardiography around the intervention It shows the (A) eccentric location of the atrial septal defect (arrow), (B) appearance of the pericardial effusion before plugging the defect (arrows), (C) plugging of the defect with the device and progressing effusion (arrows), (D) proper deployment of the device with a significant pericardial effusion (arrow).

A 45-year-old woman with atrial fibrillation was admitted for transcatheter closure of an atrial septal defect. Transesophageal echocardiography (TEE) showed an OS ASD of size 30 mm in the four-chamber view and of 21 mm in the bicaval view. The total length of the interatrial septum was 6 cm. The defect had a deficient retro-aortic rim; the superior vena cava (SVC) rim was 8 mm. Therefore, the defect was considered eccentric, with the location of the defect seen in relation to the length of the interatrial septum (Figure 1A). The pulmonary veins were draining into the left atrium without any turbulent flow as seen in TEE. The right ventricular systolic pressure was 41 mmHg. The coronary angiogram was normal. Informed consent was taken and the Heart team was consulted.

**Figure 2:**
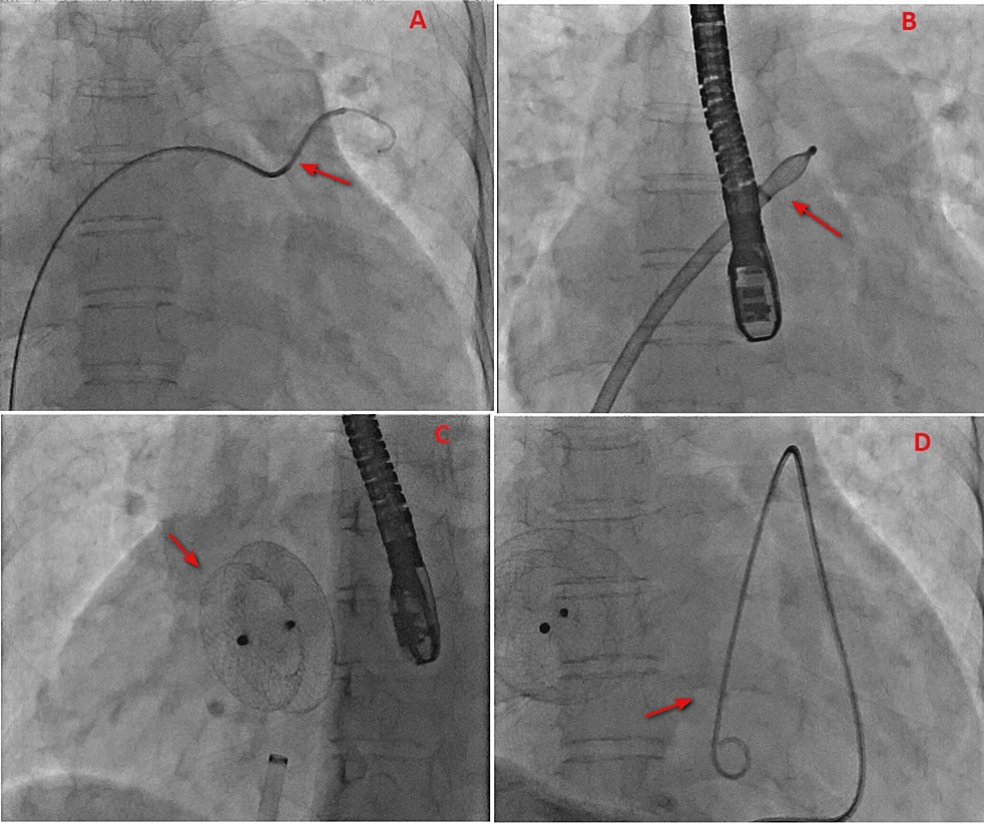
Fluoroscopy during the procedure Images show the (A) unusual course of the Amplatzer extra-stiff guide wire to the left upper pulmonary vein because of the eccentric location of the defect (arrow), (B) partial release of the left atrial disc in the left atrium rather than the upper pulmonary vein (arrow), (C) immediate perfect position of the device after releasing it (arrow) and (D) stable device position during pericardial tapping using pigtail aspiration (arrow).

The defect was tried to plug through femoral access using a 34-mm Lifetech ASD device (Lifetech Scientific Co., Shenzhen, China) using the 14-Fr dedicated sheath. The procedure was done under fluoroscopic and TEE guidance. We noticed the tortuous course of the 0.035 inch x 260 cm Terumo guide wire (Terumo Corporation, Tokyo, Japan) and 5-Fr Judkins right (JR) diagnostic catheter combo in attempting to wire the left upper pulmonary vein, which was mostly due to the cranial position of the catheter more towards the junction of SVC and right atrium (SVC-RA) because of the eccentric defect, i.e., the interatrial septal length of 60 mm, 30-mm defect and 8-mm SVC rim (Figure 2A). The 14-Fr sheath and its dilator were advanced from the groin till the combo reached the level of the hepatic vein in the inferior vena cava. The dilator was exchanged with a 5-Fr JR diagnostic catheter, and this combo was advanced into the left atrium over the Terumo guide wire in the place of the extra-stiff Amplatzer guide wire (Boston Scientific, Marlborough, MA) to avoid injury. It was noticed in TEE that the combo of the 14-Fr sheath and the diagnostic 5-Fr was not at all through the centre of the defect. Therefore, the upper end of the delivery sheath was parked in the left atrium before loading the ASD device into the delivery sheath. The release of the left atrial disc was initiated in the left atrium rather than the left upper pulmonary vein (Figure 2B). Despite this precaution, we noticed pericardial effusion before plugging the defect probably due to the SVC-RA junction injury because of the more cranial position of the sheath and the partially released left atrial disc close to the SVC-RA junction (Figure 1B). The device seated properly across the ASD when it was pulled down across the defect (Figures 1C, 1D, 2C). Pericardial effusion progressed to a depth of 10 mm within one hour after device implantation (Figure 1D). A pigtail aspiration was initiated (Figure 2D).

**Figure 3:**
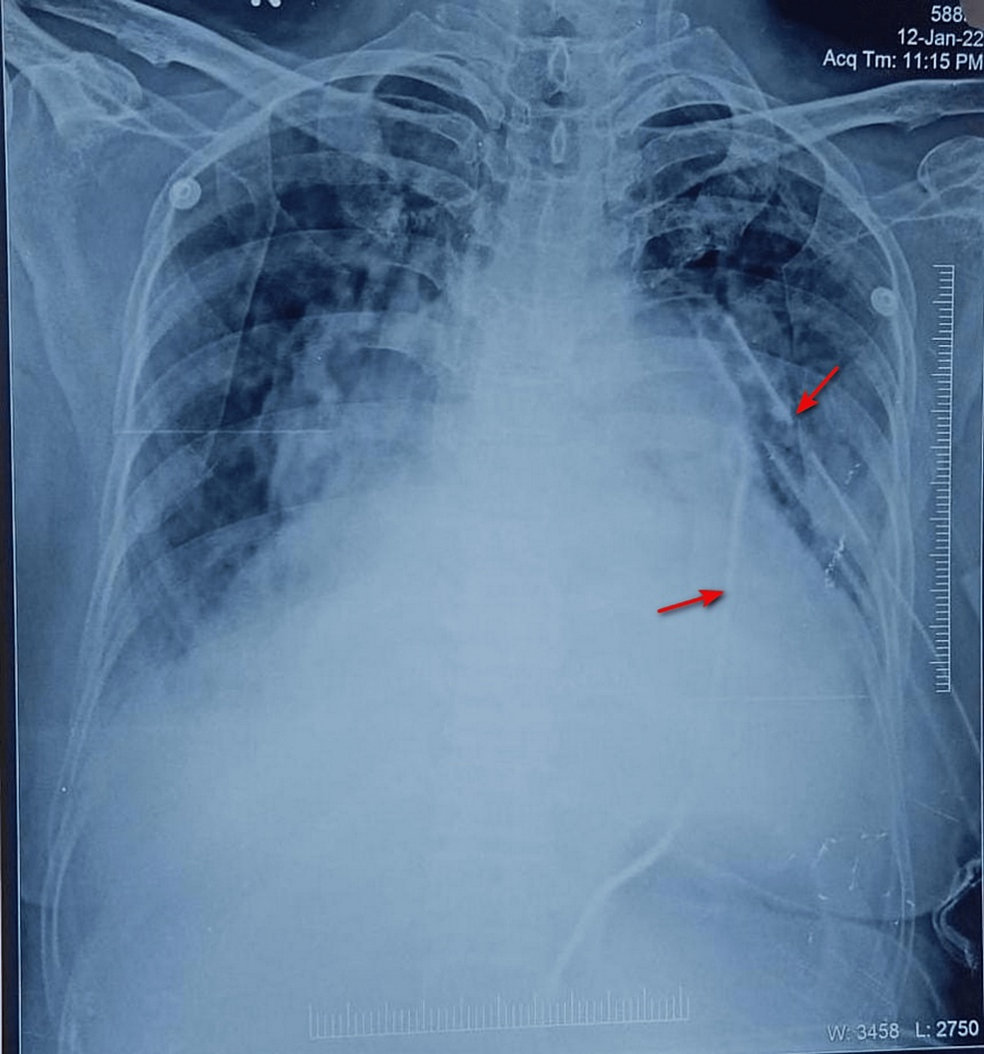
Chest X-ray after transcatheter closure of the atrial septal defect The anteroposterior view shows the intercostal drainage tube in the pleural cavity (right arrow) and the pigtail catheter in the pericardial cavity (left arrow).

A follow-up contrast-enhanced computed tomography (CECT) scan performed the next morning revealed a contained tear of 2.1 cm in the SVC-RA junction, and the left upper pulmonary vein was noticed to be tortuous without any apparent injury. One unit of fresh blood was given. One gram tranexamic acid was given IV followed by 500 mg every eight hours for the next two days. We had a second aspiration of 400 ml after 36 hours and third aspiration of 200 ml after 48 hours. The CECT showed significant left-sided pleural effusion that was detected when the patient was evaluated for room air desaturation on the second day of the procedure. The insertion of intercostal drainage tube showed 400 ml of the haemorrhagic effusion that reduced to nil after another 48 hours (Figure 3). The right ventricular failure and elevated right ventricular pressure was managed by diuretics, digoxin, and pulmonary vasodilators. She was successfully discharged on the sixth day after transcatheter closure. This patient has completed the six-month follow-up and is asymptomatic. Now, she is on metoprolol succinate, digoxin, and rivaroxaban 10 mg for atrial fibrillation.

Balloon sizing of the defect and left atrial appendage closure were not performed in this case because of financial constraints.

## Discussion

When an atrial defect is large, has rim deficiency, is fenestrated, and there are multiple atrial septal defects in the same patient, the defect is called a complex atrial septal defect that poses challenges during transcatheter closure. Malzahn et al. performed device closure of the large atrial defects in consecutive 275 patients [6]. The incidence of periprocedural complications was noticed in 20.4% cases and pericardial effusion was noticed in 5.5% cases. In an adult, an ASD that is bigger than 25 mm in size is called a large defect [7]. The device is called large when the device size is larger than 30 mm; the rims are called adequate when they are at least 5 mm. In our case, the retro-aortic rim was deficient, which is acceptable for transcatheter closure in most of the studies. Some of the complications like arrhythmia, pericardial effusion, device embolization, cardiac perforation, cardio-embolic stroke, and haemoglobinuria are immediate. Timely diagnosis of such rare complications using 2D echo, TEE or CECT is required for proper management by a conservative or surgical approach as observed in a study by Batta et al. [8]. Thanopoulos et al. in a study showed that ASDs with isolated rim deficiency can be safely closed without any significant complications if proper techniques are used [9]. Device closure of an ASD with complex anatomy is possible using several modified techniques [5]. The stiff sheath with dilator negotiation in such situations may cause cardiac perforation in the cases of larger defects, as in our case, which need close monitoring during the procedure using both TEE and fluoroscopy [10]. There may be cardiac perforation during sheath negotiation of anchoring the device across the defect. An abnormal course of the guide wire during parking the extra-stiff guide wire in the left upper pulmonary vein may cause injury to the pulmonary vein that may manifest as a pleural effusion as in our case [6]. However, close monitoring and appropriate conservative management may sometimes save the patient undergoing cardiac surgery [11]. Although it is rare to encounter remote cardiac erosion despite the successful closure of an atrial septal defect, regular follow-up of the patients is essential as observed in a study by Zhang and Ding [12].

## Conclusions

Device closure of an eccentric and large atrial septal defect may be challenging. Patients should be closely monitored during and after the procedure. Many a time minor cardiac perforation can be managed conservatively or by surgery without explanting the device if device alignment is proper.

## References

[1] Stout KK, Daniels CJ, Aboulhosn JA (2019). 2018 AHA/ACC Guideline for the management of adults with congenital heart disease: a report of the American College of Cardiology/American Heart Association Task Force on clinical practice guidelines. J Am Coll Cardiol.

[2] Le Gloan L, Legendre A, Iserin L, Ladouceur M (2018). Pathophysiology and natural history of atrial septal defect. J Thorac Dis.

[3] Takaya Y, Akagi T, Nakagawa K (2020). Clinical significance of septal malalignment for transcatheter closure of atrial septal defect. J Interv Cardiol.

[4] Jung SY, Kim AY, Jung JW, Choi JY (2019). Procedural, early and long-term outcomes after percutaneous closure of atrial septal defect: comparison between large and very large atrial septal defect groups. Korean Circ J.

[5] Jung SY, Choi JY (2018). Transcatheter closure of atrial septal defect: principles and available devices. J Thorac Dis.

[6] Malzahn L, Bertog S, Sievert K (2022). Transcatheter closure of large atrial septal defects in adults. (Article in press). Cardiovasc Revasc Med.

[7] Thanopoulos BV, Soendergaard L, Ngugen HL (2021). International experience with the use of Cocoon septal occluder for closure of atrial septal defects. Hellenic J Cardiol.

[8] Batta A, Naganur S, Rajan A, Ary KA, Gawalkar A, Barwad P (2021). Retrieval and repositioning of an embolized atrial septal defect closure device using a gooseneck snare. Egypt Heart J.

[9] Savis A, Simpson J (2018). Echocardiographic approach to catheter closure of atrial septal defects: patient selection, procedural guidance and post-procedural checks. Echo Res Pract.

[10] Zhu P, Qiang H, Liu F, Xie P, Zheng S, Sun Y (2020). Clinical evaluation of percutaneous and intra-operative device closure of atrial septal defects under transesophageal echocardiographic guidance: one center experience and mid-term follow-up. J Cardiothorac Surg.

[11] Everett AD, Jennings J, Sibinga E (2009). Community use of the amplatzer atrial septal defect occluder: results of the multicenter MAGIC atrial septal defect study. Pediatr Cardiol.

[12] Zhang ZQ, Ding JW (2021). Perforation of the atrial wall and aortic sinus after closure of an atrial septal defect with an Atriasept occluder: a case report. J Cardiothorac Surg.

